# Diagnosis of ankylosing spondylitis: A bibliometric review from 2008 to 2024

**DOI:** 10.1097/MD.0000000000050931

**Published:** 2026-10-02

**Authors:** Zhihao Qiu, Ruibo Xia, Keming Chen, Zhe Yang, Zijie Chen, Kepeng Yang

**Affiliations:** a The Second School of Clinical Medicine, Zhejiang Chinese Medical University, Hangzhou, Zhejiang, China; b Department of Rheumatology and Immunology, The Second Affiliated Hospital of Zhejiang Chinese Medical University, Hangzhou, Zhejiang, China; c The First School of Clinical Medicine, Zhejiang Chinese Medical University, Hangzhou, Zhejiang, China.

**Keywords:** ankylosing spondylitis, axial spondyloarthritis, bibliometrics, biomarkers, diagnosis, magnetic resonance imaging, Mendelian randomization analysis

## Abstract

**Background:**

The pathogenesis and pathological progression of ankylosing spondylitis (AS) remain unclear. Owing to the absence of a gold standard for diagnosing AS, diagnostic delays are frequently encountered in clinical practice, exacerbating the disease burden. In the past 16 years, several diagnostic studies on AS have been published. This study aimed to summarize the developmental trends and frontier hotspots in AS diagnosis through a bibliometric review.

**Methods:**

Data were extracted from English publications on AS diagnosis from the Web of Science Core Collection (January 1, 2008–October 21, 2024). CiteSpace, VOSviewer, and the Biblioshiny R package were used to analyze the overview of the publication output, countries/regions, institutions, authors, sources, references, and keywords.

**Results:**

A total of 1205 articles from 396 journals across 65 countries were included. The highest annual output occurred in 2021 (n = 111, 9.21%), whereas 2015 had the highest average number of citations (n = 5.1). *Clinical Rheumatology* (n = 76, 6.31%) was the most influential journal. China (n = 244, 20.25%), Berlin Institute of Health (n = 116, 9.63%), and Sieper J (n = 28, 2.32%) ranked first in terms of country, institution, and author productivity, respectively. The top 3 reference clusters were “diagnostic criteria,” “therapeutics,” and “recommendations.” Burst detection identified 4 emerging post-2021 keywords: “children,” “cells,” “spondyloarthritis,” and “expression.”

**Conclusion:**

AS diagnosis research has evolved through a growth phase (2008–2021), followed by a transitional or reorientation phase (2022–2024). Magnetic resonance imaging, Mendelian randomization, and biomarkers are current hotspots; Mendelian randomization serves as an etiological adjunct with the potential to inform biomarker discovery, though its findings do not yet constitute diagnostic tools. The apparent slowdown in recent years largely reflects evolving terminology (AS vs radiographic axial spondyloarthritis) and bibliometric time-lag bias rather than true intellectual stagnation, alongside fragmented pathophysiological insights and a lack of specific clinical features.

## 1. Introduction

Axial spondyloarthritis (ax-SpA) refers to a group of chronic immune-mediated disorders predominantly affecting the axial joints. It is characterized by inflammatory low back pain as the primary clinical symptom.^[1]^ Radiographic ax-SpA is defined as ankylosing spondylitis (AS).^[2]^ The pathogenesis of AS remains incompletely understood, although genetic factors, such as human leukocyte antigen B27 (HLA-B27), are strongly associated.^[3]^ Other genetic contributors include IL-23R, ERAP1, and non-B27 major histocompatibility complex class I genes.^[4]^ Nongenetic factors such as gut microbiota also play a role in AS development.^[5]^ The pathological progression of AS is complex. Historically, enthesitis was considered the initiating factor in AS pathology.^[6]^ However, the failure of interleukin-23-targeted therapies to suppress enthesitis has prompted researchers to consider whether spinal inflammation might exhibit a persistent inflammatory mechanism independent of interleukin-23.^[7]^ For instance, some studies have proposed that bone marrow lesions are key lesions driving the most characteristic features of AS.^[8]^

Given that the pathogenesis and pathological progression of AS remain unclear, there is currently no gold standard for its diagnosis. The primary diagnostic criteria in use are the revised New York criteria and the classification criteria for ax-SpA established by the Assessment of SpondyloArthritis International Society (ASAS). In 2009, ASAS reported that with imaging evidence, the sensitivity and specificity of the ax-SpA classification criteria were 66.2% and 97.3%, respectively.^[2]^ In 2017, a meta-analysis showed that the “imaging arm only” ASAS classification criteria had a sensitivity of 30% and specificity of 97%.^[9]^ Delayed diagnosis due to underdiagnosis imposes substantial burdens on patients, exacerbating physical damage, increasing healthcare costs, and diminishing quality of life, including psychological impacts.^[10]^

Bibliometric review studies publications in a specific research field from both quantitative and qualitative perspectives. Visualization technology displays the structure, patterns, and distribution of knowledge graphs in this research field. For this study, analysis software was used to conduct a bibliometric review of relevant countries, authors, journals, and keywords in a specific research field to obtain the research status, hotspots, and development trends. Some scholars have conducted bibliometric analyses on clinical trials and ectopic ossification of AS,^[11,12]^ but there is no bibliometric review on the diagnosis of AS-related fields. Therefore, this study aimed to review the diagnostic research on AS published in the past 16 years, conduct visual analysis to comprehensively understand the research trends in this field, and explore current research hotspots and potential challenges in diagnosing AS.

## 2. Materials and methods

### 2.1. Data sources and retrieval strategies

Regarding data sources and literature screening criteria, our team referenced methodologies from prior bibliometric studies and selected the Web of Science Core Collection (WoSCC) as the database. English-language “articles” and “reviews” were chosen as publication type filters. As noted earlier, the classification criteria for AS were established in 2009, a critical milestone. To avoid missing studies foundational to diagnostic classification, the search timeframe was expanded by 1 year, spanning from January 1, 2008, to the search date. For search strategies, while most bibliometric analyses use “topic”-based queries, this study adopted a “title + abstract + keyword” format to exclude potential biases from “keyword plus” indexing. The specific content is as follows: (TI=((“Spondylitis, Ankylosing” or “Bechterew’s Disease” or “Bechterews Disease” or “Marie-Struempell Disease” or “Marie Struempell Disease” or “Spondylarthritis Ankylopoietica” or “Rheumatoid Spondylitis” or “Spondylitis, Rheumatoid” or “Ankylosing Spondylitis” or “Ankylosing Spondylarthritis” or “Ankylosing Spondylarthritides” or “Spondylarthritides, Ankylosing” or “Spondylarthritis, Ankylosing” or “Ankylosing Spondyloarthritis” or “Ankylosing Spondyloarthritides” or “Spondyloarthritides, Ankylosing” or “Spondyloarthritis, Ankylosing” or “Spondylitis Ankylopoietica” or “Bechterew Disease” or “Spondyloarthritis Ankylopoietica”) and (“diagnosis”)) or AB=((“Spondylitis, Ankylosing” or “Bechterews Disease” or “Bechterews Disease” or “Marie-Struempell Disease” or “Marie Struempell Disease” or “Spondylarthritis Ankylopoietica” or “Rheumatoid Spondylitis” or “Spondylitis, Rheumatoid” or “Ankylosing Spondylitis” or “Ankylosing Spondylarthritis” or “Ankylosing Spondylarthritides” or “Spondylarthritides, Ankylosing” or “Spondylarthritis, Ankylosing” or “Ankylosing Spondyloarthritis” or “Ankylosing Spondyloarthritides” or “Spondyloarthritides, Ankylosing” or “Spondyloarthritis, Ankylosing” or “Spondylitis Ankylopoietica” or “Bechterew Disease” or “Spondyloarthritis Ankylopoietica”) and (“diagnosis”)) or AK=((“Spondylitis, Ankylosing” or “Bechterews Disease” or “Bechterews Disease” or “Marie-Struempell Disease” or “Marie Struempell Disease” or “Spondylarthritis Ankylopoietica” or “Rheumatoid Spondylitis” or “Spondylitis, Rheumatoid” or “Ankylosing Spondylitis” or “Ankylosing Spondylarthritis” or “Ankylosing Spondylarthritides” or “Spondylarthritides, Ankylosing” or “Spondylarthritis, Ankylosing” or “Ankylosing Spondyloarthritis” or “Ankylosing Spondyloarthritides” or “Spondyloarthritides, Ankylosing” or “Spondyloarthritis, Ankylosing” or “Spondylitis Ankylopoietica” or “Bechterew Disease” or “Spondyloarthritis Ankylopoietica”) and (“diagnosis”))). In the analyses below, reviews and original articles were retrieved together and analyzed as a combined corpus; no separate sub-analysis by article type (e.g., systematic review and meta-analysis, narrative review) was undertaken. To evaluate the robustness of our findings to the incomplete 2024 data year and the evolving “AS versus axial spondyloarthritis” nomenclature, 2 sensitivity analyses were performed, as described in the Results. We elected to retain “ankylosing spondylitis” (AS) as the anchoring bibliometric construct for 3 reasons. First, AS is a stable, continuously indexed term throughout the entire 2008 to 2024 window, whereas “axial spondyloarthritis” entered widespread use only after the 2009 ASAS criteria, so an ax-SpA anchor would systematically omit early-period literature and break the longitudinal comparability essential to a trend analysis. Second, the 2009 ASAS criteria define AS as synonymous with radiographic ax-SpA, so the 2 labels denote the same population under different names rather than distinct diseases. Third, anchoring on AS tightly bounds the review to the question of “diagnosis of ankylosing spondylitis” specifically, avoiding conflation with the broader spondyloarthritis spectrum. We acknowledge that this choice undercaptures post-2010 studies that favor the newer terminology, and we therefore quantified and interpreted this limitation through the sensitivity analysis described in the Results. For the sensitivity analysis, the expanded (union) search additionally included the following non-ankylosing ax-SpA terms in the same title, abstract, and author keywords fields: “Axial Spondyloarthritis,” “Axial Spondyloarthritides,” “Non-Radiographic Axial Spondyloarthritis,” “Nonradiographic Axial Spondyloarthritis,” “AxSpA,” “axSpA,” “r-axSpA,” and “nr-axSpA” combined with “diagnosis.” Two records re-dated by the database to 2025 after the October 21, 2024, search date (online-first articles) were excluded from the final counts, which therefore include publication years 2008 to 2024 only.

### 2.2. Data extraction and analysis

To enhance the visualization of the results, this study employed a multi-software approach for bibliometric review. CiteSpace (6.4.R1), developed by Dr Chaomei Chen at Drexel University, was used for network mapping, cluster analysis, and burst detection. This tool facilitated the analysis of keywords, citations, and dual-map overlays of journals to identify research hotspots, frontiers, and temporal trends. VOSviewer (v1.6.20), created by Nees Jan van Eck and Ludo Waltman at Leiden University, was used to construct visual collaboration networks among institutions and authors, identifying leading contributors in the field. Additionally, the Biblioshiny R package (built on R 4.3.3), an open-source tool developed by Aria and Cuccurullo, integrated bibliometric network analysis and visualization to map the knowledge structure of the target literature.^[13]^ Compared with the aforementioned tools, the “bibliometrix” package in R (Massimo Aria and Corrado Cuccurullo, University of Naples Federico II, Italy) offers a more comprehensive suite of techniques, enabling annual citation analysis, core journal identification, country/region assessments, and keyword/topic correlations. Figure 1 illustrates the publication screening process, bibliometric workflow, and integration of analytical software.

**Figure 1.**
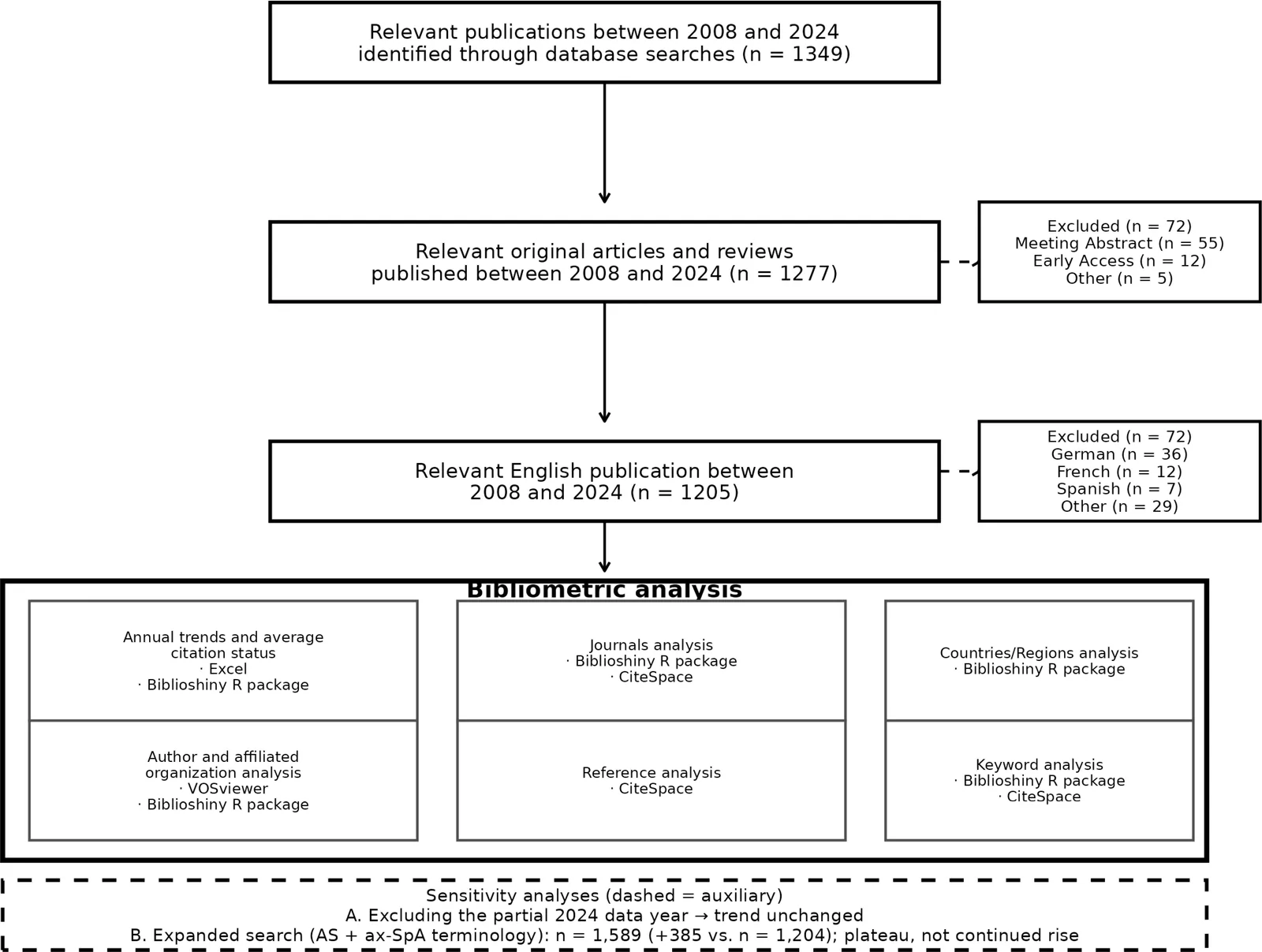
The inclusion and exclusion of publications on AS and the bibliometric workflow. The dashed box indicates the 2 sensitivity analyses (exclusion of the partial 2024 data year and the expanded axial spondyloarthritis terminology search), which are further detailed in Figure 8. AS = ankylosing spondylitis.

### 2.3. Ethics statement

This study analyzed only published, publicly available bibliometric data extracted from the WoSCC and did not involve human participants, human material, or identifiable personal data. Ethical approval and informed consent were therefore not required, and the need for review was waived.

## 3. Results

### 3.1. Annual trends and average citations of publications

From January 1, 2008, to October 21, 2024, a total of 1205 AS diagnosis-related articles were published in the WoSCC. As shown in Figure 2A, the annual publication output and trend curve (*R*^2^ = 0.9708) indicate a peak in 2021 (n = 111), followed by a gradual decline in subsequent years. Figure 2B illustrates the average annual citation count for this field, which remained relatively stable overall, with the highest average number of citations recorded in 2015 (n = 5.1). Because 2024 was only partially recorded at the time of the search (through October 21), a sensitivity analysis excluding that incomplete year was performed and did not alter the overall trend (see Sensitivity analyses).

**Figure 2.**
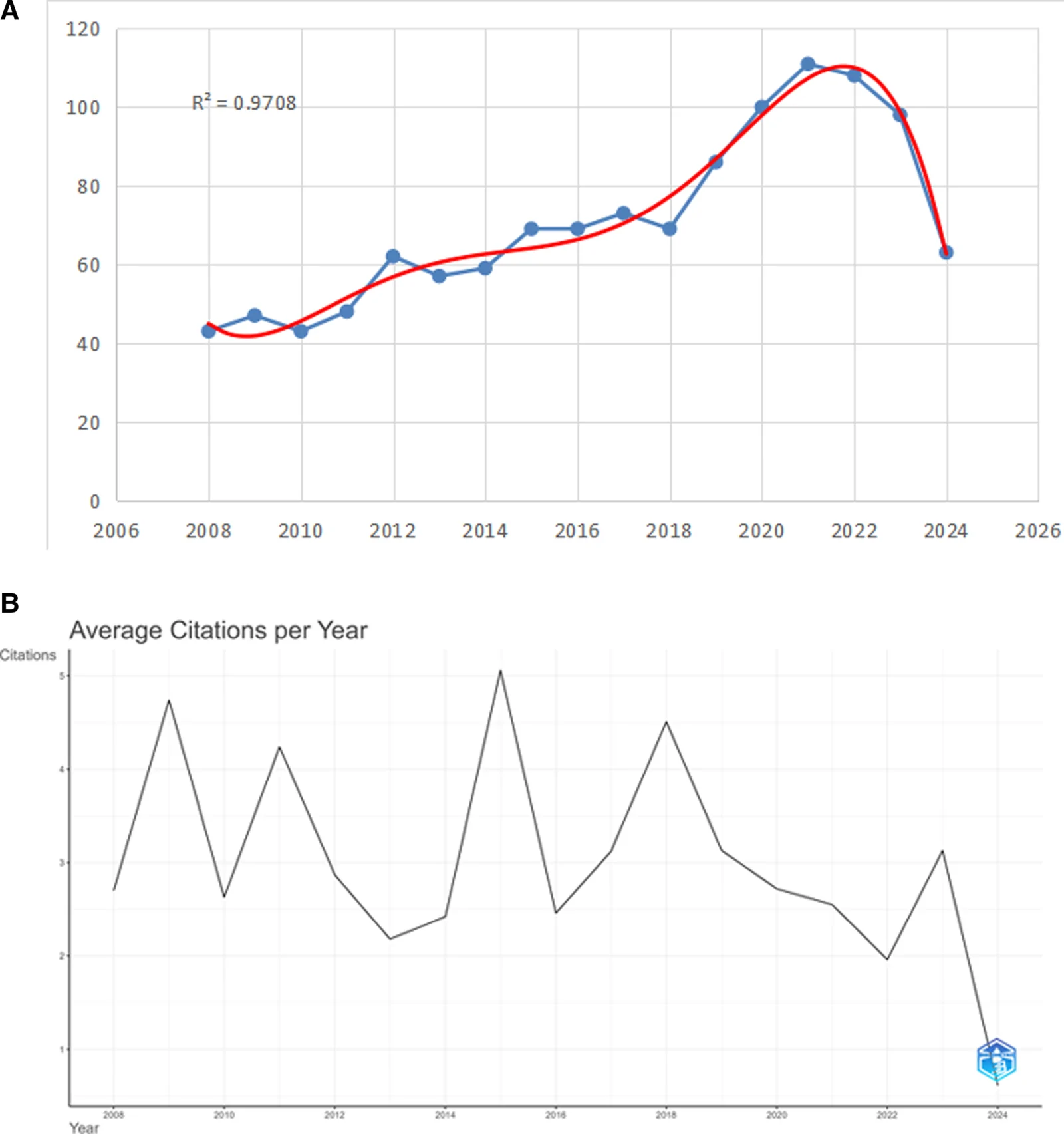
Publication output and citation trends of AS diagnosis-related research. (A) The annual publication volume and trend curve of AS diagnosis-related articles from 2008 to 2024 (*R*^2^ = coefficient of determination). (B) The average yearly citation frequency of AS diagnosis-related articles over the same period, with the highest average recorded in 2015. AS = ankylosing spondylitis.

### 3.2. Journal analysis

Over the 17-year period, a total of 1205 publications related to AS diagnosis were published across 396 journals. Table 1 lists the top 10 most influential journals, reporting their H-index, G-index, M-index, total citations (TC), number of publications, and year of first publication (PY-start). The H-index denotes that at least *h* articles were cited *h* times each, with the remaining articles cited no more than *h* times. The G-index indicates that the average number of citations of the top *g* articles is at least *g*. The M-index is calculated by dividing the H-index by the number of years since the first publication. Figure 3A illustrates core journals based on Bradford’s Law, which posits that a majority of publications in a specific field are concentrated in a small number of journals. Within AS diagnosis research, 12 journals were identified as core, with *Clinical Rheumatology* being the most influential, publishing 76 articles (6.31% of total output) over 16 years. Figure 3B presents a dual-map overlay of journals, where left-side journals represent citing sources and right-side journals represent cited targets. Lines denote citation relationships. Three primary citation pathways were identified: articles published in Molecular/Biology/Genetics, Health/Nursing/Medicine, and Sports/Rehabilitation/Sport journals were most frequently cited by Medicine/Medical/Clinical journals.

**Table 1:** The top 10 most influential journals.

Source	H-index	G-index	M-index	TC	NP	PY-start
*Annals of the Rheumatic Diseases*	23	28	1.353	3773	28	2008
*Clinical Rheumatology*	23	39	1.353	1789	76	2008
*Journal of Rheumatology*	23	34	1.353	1267	43	2008
*Arthritis and Rheumatism*	7	7	0.412	1007	7	2008
*Arthritis Research & Therapy*	16	27	1	923	27	2009
*Lancet*	2	2	0.143	863	2	2011
*Rheumatology International*	17	26	1	802	52	2008
*Inflammatory Bowel Diseases*	3	3	0.176	700	3	2008
*Clinical and Experimental Rheumatology*	16	25	0.941	674	33	2008
*Arthritis Care & Research*	12	20	0.8	568	20	2010

NP = number of publications, PY-start = year of first publication, TC = total citations.

**Figure 3.**
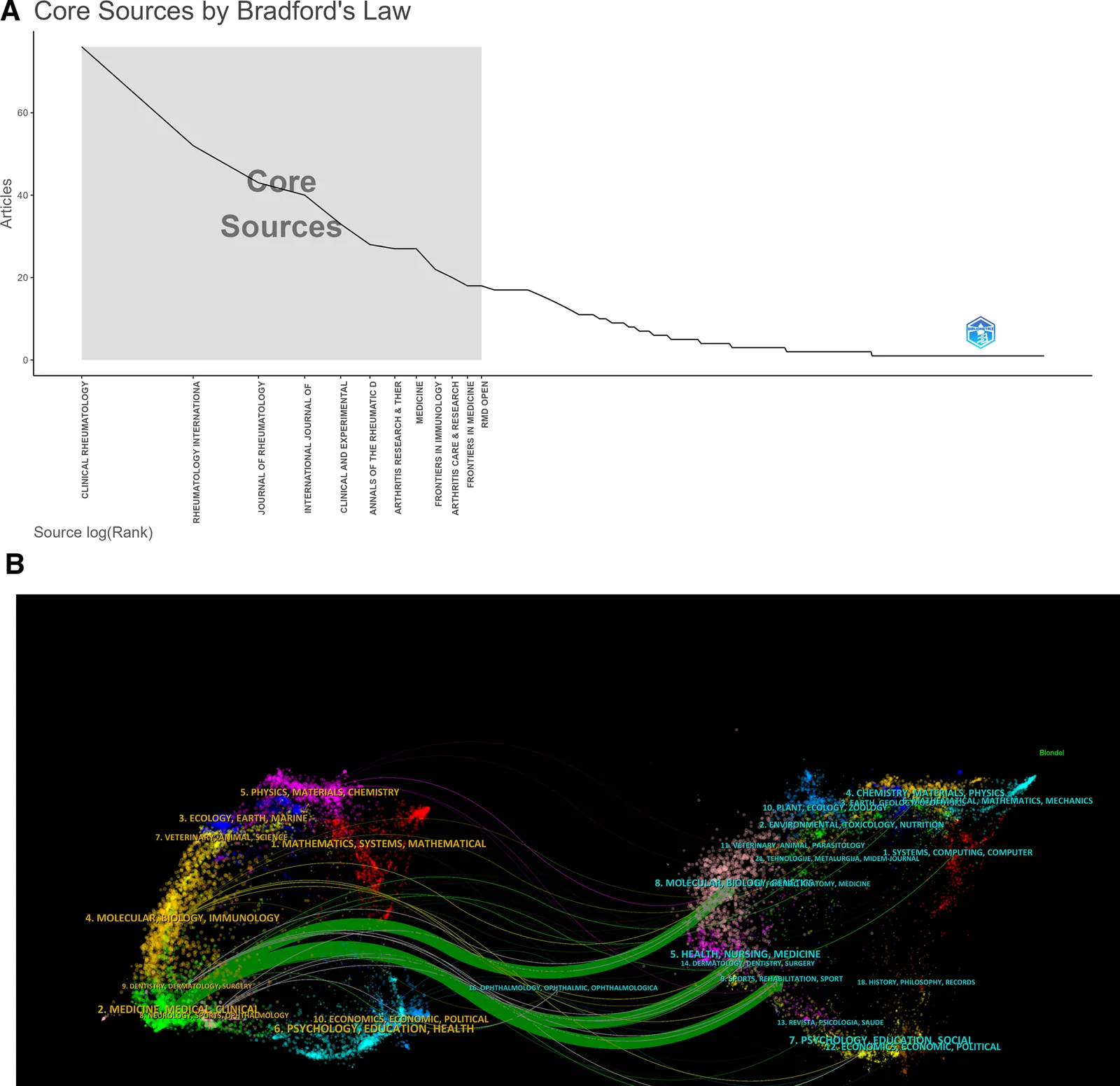
Core journals and journal citation patterns in AS diagnosis research. (A) The core journals identified by Bradford’s Law. (B) A dual-map overlay of journals, in which left-side journals represent citing sources, right-side journals represent cited targets, and lines denote citation relationships. AS = ankylosing spondylitis.

### 3.3. Country/region analysis

Table 2 reports the top 10 most productive countries by publication count, including their multiple-country publications, TC, and average article citations. China ranked highest in productivity (n = 244, 20.25%), followed by the USA (n = 183, 15.19%) and Turkey (n = 94, 7.80%). Among these top 10 countries, the USA had the most articles with international co-authorship (n = 36), followed by Germany (n = 28) and China (n = 26). In terms of overall research impact, the USA led in TC (n = 4874), followed by Germany (n = 4529) and China (n = 2621). Regarding average article quality, Germany achieved the highest average article citations (n = 67.60), followed by France (n = 39.30) and the UK (n = 30.90). To further explore international collaboration trends, a country collaboration map was generated based on author affiliations (Fig. 4A), revealing close cooperation among China, the USA, and Western European nations. The distribution of corresponding authors’ countries and their collaboration types is illustrated in Figure 4B, which shows that China had the highest total number of publications (predominantly single-country publications, or SCP), whereas countries such as the USA, Germany, and the UK exhibited higher proportions of multiple-country publications. The citation impact of these leading countries is further assessed in Figure 4C, which corroborates that the USA and Germany occupied the dominant positions with 4874 and 4529 total citations, respectively. In addition, the annual publication production over time is depicted in Figure 4D, illustrating China’s sharp and continuous upward trajectory, particularly after 2016, while the USA maintained steady growth, and countries such as the UK and Turkey exhibited relatively slower increases.

**Table 2:** Single-country publications (SCP) and multiple-country publications (MCP).

Country	Articles	Articles, %	SCP	MCP	MCP, %
China	244	20.2	218	26	10.7
USA	183	15.2	147	36	19.7
Turkey	94	7.8	92	2	2.1
Germany	67	5.6	39	28	41.8
United Kingdom	55	4.6	32	23	41.8
Italy	51	4.2	47	4	7.8
Canada	40	3.3	24	16	40
France	39	3.2	34	5	12.8
Korea	39	3.2	37	2	5.1
Spain	39	3.2	33	6	15.4

**Figure 4.**
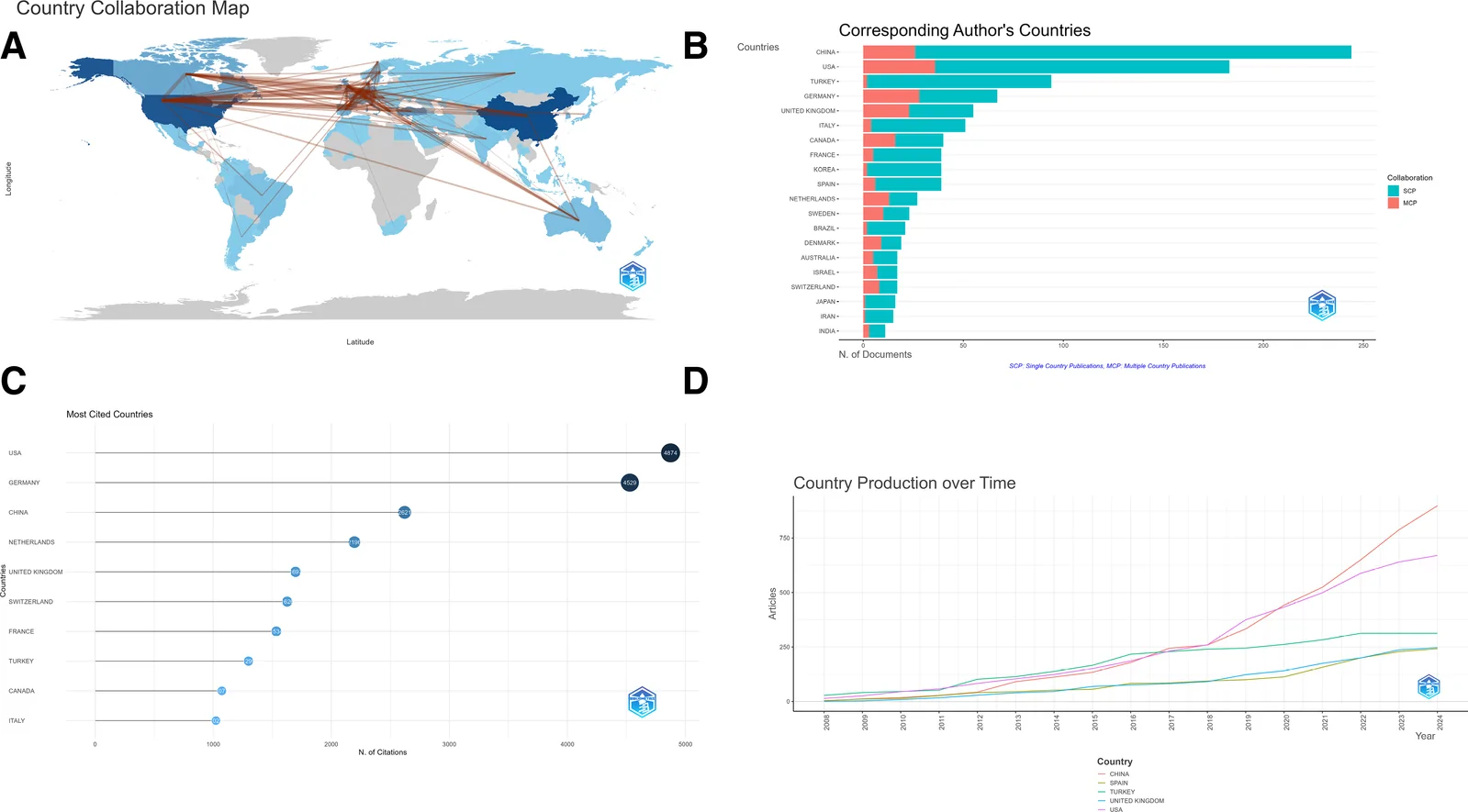
Country-level collaboration and cumulative publication output in AS diagnosis research. (A) The country collaboration network of AS-related diagnostic research publications based on author affiliations. (B) The distribution of corresponding authors’ countries and their collaboration types (SCP and MCP). (C) The most cited countries of AS-related diagnostic research publications. (D) The cumulative publication curve by country of publication origin. AS = ankylosing spondylitis, MCP = multiple-country publications, SCP = single-country publications.

### 3.4. Author and affiliated institution analysis

A total of 6056 authors from 2130 institutions have published articles in the field of AS diagnosis. The top 10 most productive institutions and authors are listed in Table 3. Among institutions, the Berlin Institute of Health (n = 116, 9.63%) was the most prolific, followed by the University of Toronto (n = 100, 8.30%) and Free University of Berlin (n = 62, 5.15%). Of the top 10 institutions, 4 were based in Germany, 2 in the USA, 2 in France, and 1 in China. Figure 5A maps institutional collaborations (>5 publications), showing 7 tightly connected clusters and 4 loosely linked clusters, with China Medical University demonstrating the highest collaboration centrality (total link strength = 50). Regarding individual authors, the top 3 by publication count were Sieper J (n = 28, 2.32%), Braun J (n = 20, 1.66%), and Baraliakos X (n = 19, 1.58%). Figure 5B illustrates the local impact of these top 10 authors measured by the H-index, in which Sieper J achieved the highest H-index. Figure 5C further visualizes the annual publication output and total annual citations over time for the top 10 authors. Notably, the top 4 authors by cumulative publications had significant influence in 2009, while Poddubnyy and Marzo-Ortega emerged as key contributors in 2023. Furthermore, Figure 5D presents the lead-author collaboration network among authors with more than 4 publications. The analysis revealed 10 author clusters, with Liu Chong from China exhibiting the highest collaboration centrality (total link strength = 59).

**Table 3:** The top 10 most productive institutions and authors.

Rank	Affiliation	Articles	Rank	Authors	Articles
1	Berlin Institute of Health	116	1	Sieper J	28
2	University of Toronto	100	2	Braun J	20
3	Free University of Berlin	62	3	Baraliakos X	19
4	Humboldt University of Berlin	62	4	van der Heijde D	19
5	Charité – Universitätsmedizin Berlin	58	5	Poddubnyy D	16
6	Chung Shan Medical University	49	6	Deodhar A	15
7	Assistance Publique – Hôpitaux de Paris (AP-HP)	47	7	Dougados M	15
8	Harvard University	47	8	Maksymowych WP	15
9	Universite Paris Cite	42	9	Rudwaleit M	15
10	University of London	42	10	Marzo-Ortega H	13

**Figure 5.**
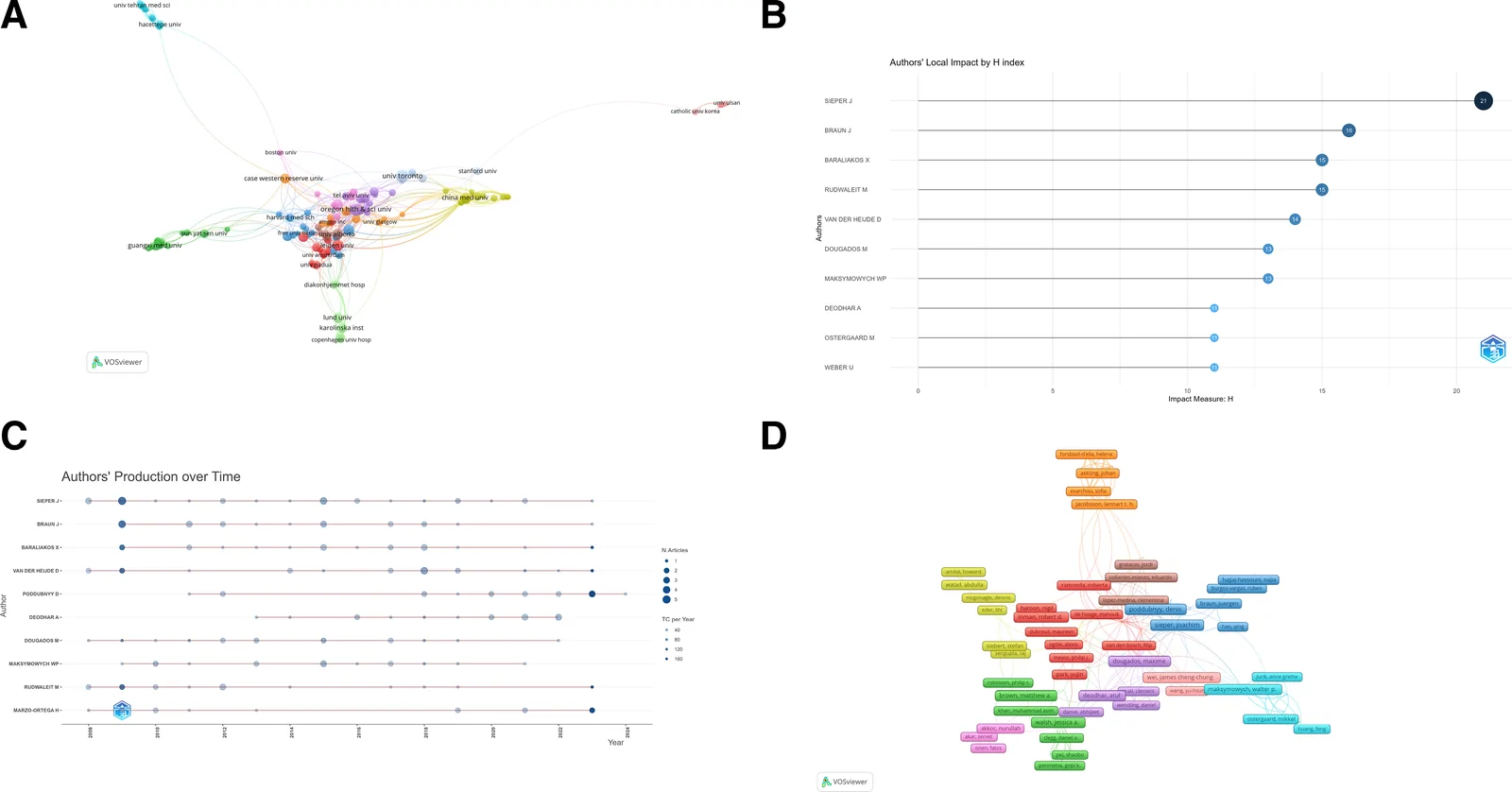
Author productivity, author collaboration, and institutional cooperation in AS diagnosis research. (A) The major institutional cooperation network (>5 publications). (B) The local impact by H-index of the top 10 most productive authors. (C) The annual output and total annual citations over time of the top 10 most productive authors. (D) The lead-author collaboration network among authors with more than 4 publications. AS = ankylosing spondylitis.

### 3.5. Reference analysis

The top 11 most-cited references are listed in Table 4 and visualized using CiteSpace (Fig. 6A). These highly cited works include the 2009 ASAS classification criteria for ax-SpA, ASAS-European League Against Rheumatism management recommendations for ax-SpA, and a 2017 Lancet review on ax-SpA, alongside studies establishing magnetic resonance imaging (MRI)-identified active sacroiliitis as a diagnostic basis for ax-SpA. To categorize these references, keyword-based citation cluster analysis was performed (Fig. 6B), revealing 12 clusters. The top 3 clusters were “diagnostic criteria,” “therapeutics,” and “recommendations.”

**Table 4:** The top 11 most-cited literature.

Frequency	Degree	Label	Author	Year	Source
67	31	Rudwaleit M (2009)	Rudwaleit M	2009	Ann Rheum Dis
54	20	van der Heijde D (2017)	van der Heijde D	2017	Ann Rheum Dis
37	7	Sieper J (2017)	Sieper J	2017	Lancet
36	31	Rudwaleit M (2009)	Rudwaleit M	2009	Ann Rheum Dis
36	20	Ward MM (2019)	Ward MM	2019	Arthritis Rheumatol
33	33	Rudwaleit M (2009)	Rudwaleit M	2009	Ann Rheum Dis
30	5	Taurog JD (2016)	Taurog JD	2016	New Engl J Med
28	22	Rudwaleit M (2011)	Rudwaleit M	2011	Ann Rheum Dis
27	21	Rudwaleit M (2009)	Rudwaleit M	2009	Arthritis Rheum-US
26	12	van der Heijde D (2011)	van der Heijde D	2011	Ann Rheum Dis
26	12	Braun J (2011)	Braun J	2011	Ann Rheum Dis

**Figure 6.**
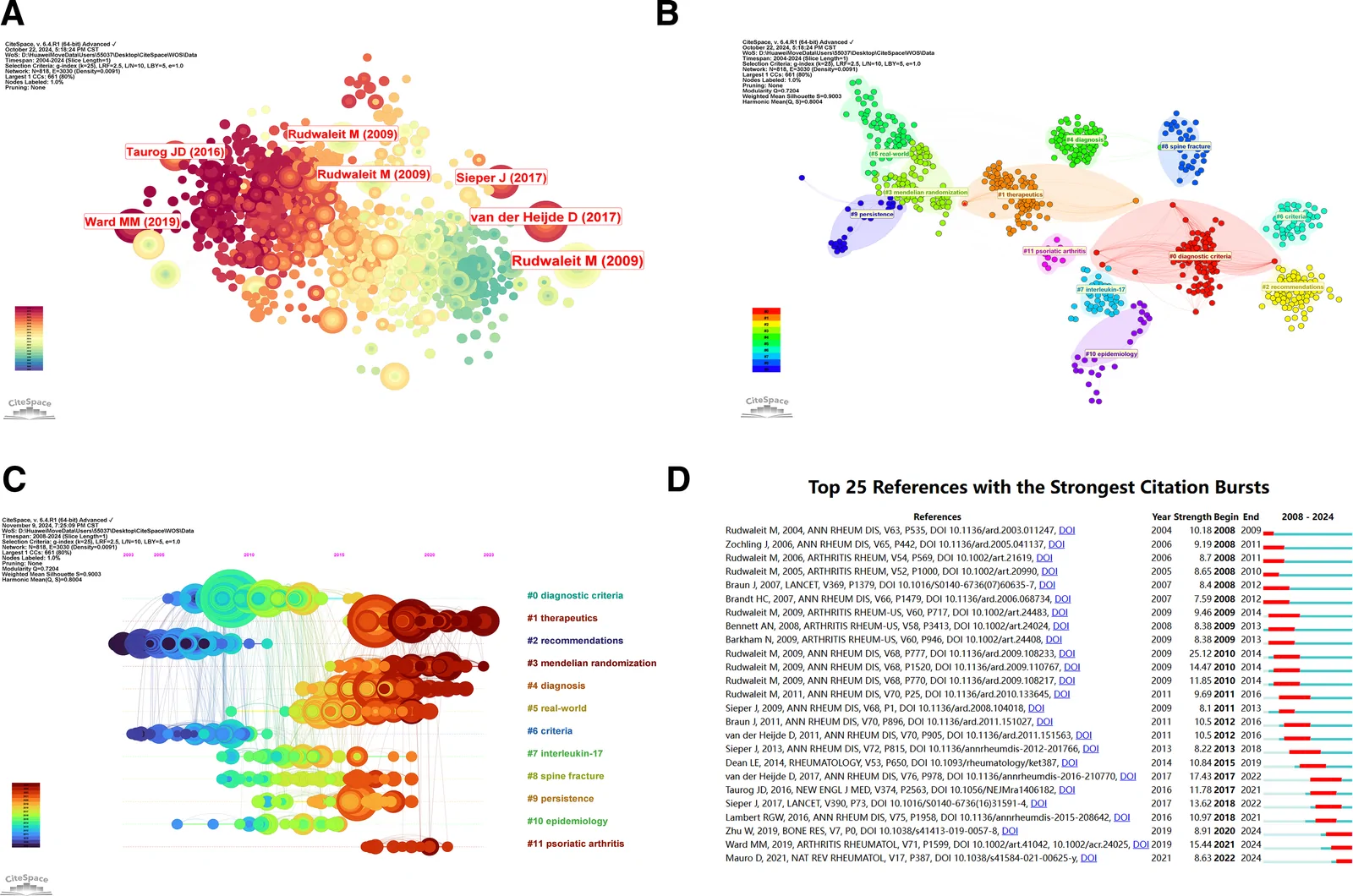
Cited references, citation clusters, and research fronts in AS diagnosis research. (A) The network of the most highly cited references in AS diagnosis research. (B) CiteSpace cluster analysis of publications related to the diagnosis of AS. (C) The timeline view of citation clusters related to the diagnosis of AS. (D) Burst detection of the most impactful publications related to the diagnosis of AS. AS = ankylosing spondylitis.

To investigate the decline in AS diagnosis-related publications post-2021, a timeline view of citation clusters was generated (Fig. 6C). Post-2021 clusters predominantly focused on “therapeutics,” “Mendelian randomization,” “diagnosis,” and “psoriatic arthritis.” A burst detection analysis (used to identify emerging research trends) further highlighted the 25 most impactful references (Fig. 6D), with parameters including the Year (first publication year), Begin/End (timeframe boundaries), and Strength (intensity of citation bursts). The results showed that the 2021 publication titled “Ankylosing spondylitis: an autoimmune or autoinflammatory disease?” has emerged as a frontier topic between 2022 and 2024.

### 3.6. Keyword analysis

The most frequent keyword over the 17-year period was “Ankylosing Spondylitis,” which appeared in 577 articles. A word cloud (Fig. 7A) displays the top 50 high-frequency keywords excluding “Ankylosing Spondylitis.” Keyword cluster analysis by subject category (Fig. 7B) identified 8 clusters, including “PHARMACOLOGY & PHARMACY,” “BIOTECHNOLOGY & APPLIED,” and “MEDICINE, RESEARCH & EXPERIMENTAL” alongside “RHEUMATOLOGY.” A Three-Field Plot integrating keywords (DE), authors (AU), and Keyword Plus (ID) (Fig. 7C) mapped the relationships between the research foundations and themes. Key DE terms included “ankylosing spondylitis,” “axial spondyloarthritis,” and “diagnosis,” while ID terms focused on “society classification criteria” and “prevalence.” To explore post-2021 publication declines, trend topics (Fig. 7D) and keyword burst detection (Fig. 7E) were analyzed. Trends in 2021 highlighted “arthritis,” “rheumatic diseases,” and “ankylosing spondylitis (AS),” shifting to “disease activity,” “biomarkers,” and “spondyloarthritis” in 2022. Burst detection identified 4 post-2021 keywords: “children,” “cells,” “spondyloarthritis,” and “expression.” Two classes of high-frequency terms warrant explicit distinction. “Magnetic resonance imaging” and “HLA-B27” index primary diagnostic research on AS itself. By contrast, “rheumatoid arthritis” and “inflammatory bowel disease” most plausibly reflect differential-diagnostic overlap – studies in which AS is examined alongside conditions sharing its clinical features – rather than AS diagnosis per se, whereas “epidemiology” maps to etiological and prevalence research.

**Figure 7.**
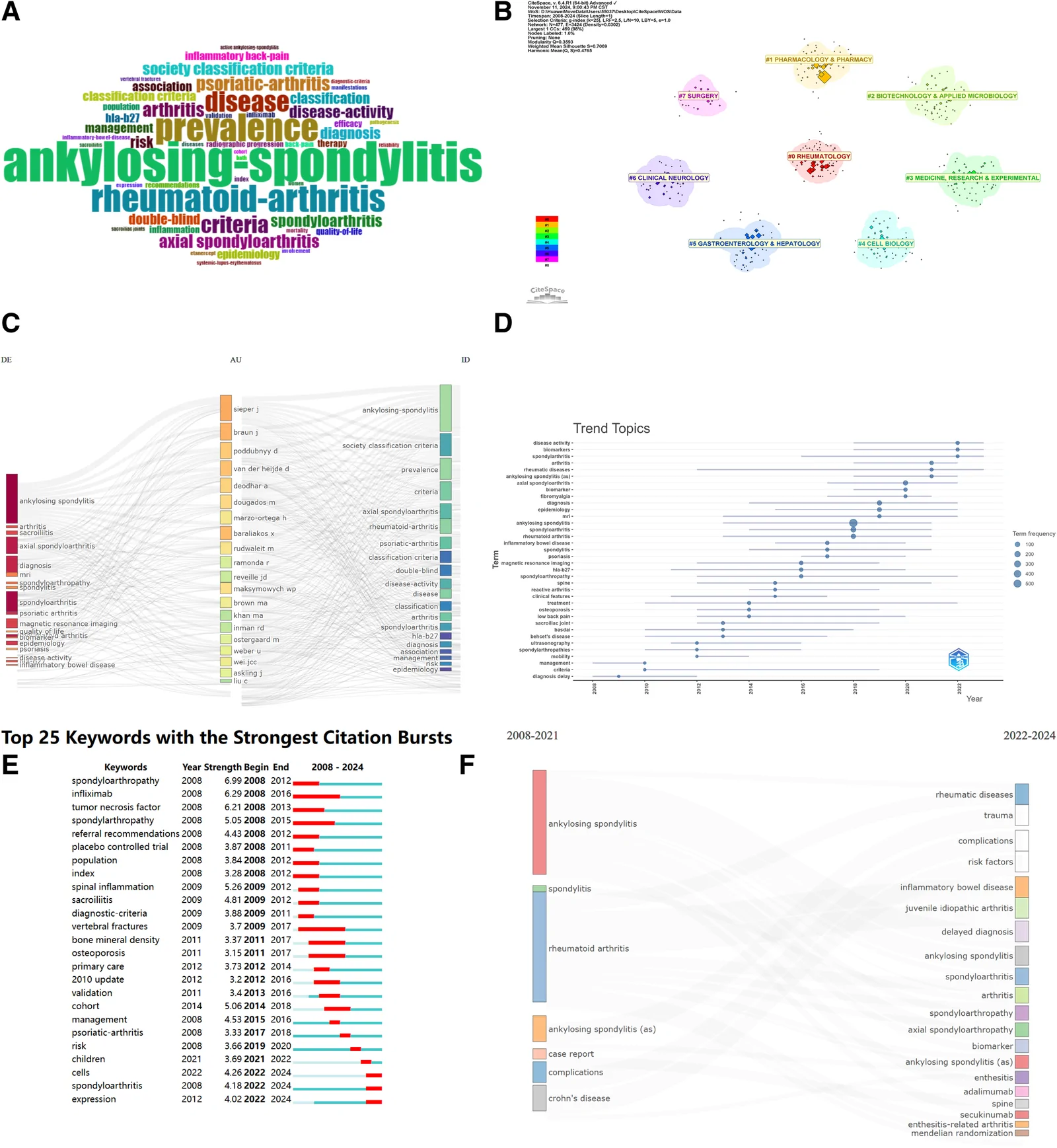
Keyword co-occurrence, clustering, and thematic evolution in AS diagnosis research. (A) A word cloud of the top 50 high-frequency keywords. (B) Keyword cluster analysis based on subject categories. (C) A Three-Field Plot integrating keywords (DE), authors (AU), and Keyword Plus (ID). (D) Trend topics based on keywords. (E) Burst detection based on keywords. (F) Topic evolution based on keywords across 2008 to 2021 and 2022 to 2024. AS = ankylosing spondylitis, AU = authors, DE = author keywords, ID = Keywords Plus.

Thematic evolution (Fig. 7F) divided the timeline into 2008 to 2021 and 2022 to 2024. The earlier phase focused on AS-rheumatoid arthritis (RA) differential diagnosis, Crohn disease complications, and case reports. Post-2021 themes diversified, expanding complications to inflammatory bowel disease, diagnostic focus to enthesitis-related arthritis and juvenile idiopathic spondyloarthritis, and therapeutic attention to adalimumab and secukinumab. Emerging topics included psychological trauma and Mendelian randomization (MR).

### 3.7. Sensitivity analyses

To further assess the robustness of our findings, 2 sensitivity analyses were performed. First, reexamining annual publication counts after excluding all 2024 records did not change the overall trend: the rise through the 2010s, the peak in the early 2020s, and the subsequent leveling-off all persisted. Second, annual output under the AS-centered search (n = 1205, 2008–2024) was compared with an expanded union search that additionally included ax-SpA terminology (AxSpA, r-axSpA, nr-axSpA, non-radiographic axial spondyloarthritis; n = 1589). The expanded search captured 384 additional publications, predominantly from 2017 onward. Whereas the AS-only search declined after its 2021 peak (111 to 66 publications by 2024), the expanded search also reached a peak in 2021 (167 publications) and then declined to 119 by 2024, but far more gently than the AS-only search (a 28.7% vs 40.5% reduction over 2021–2024). This confirms that a substantial part of the apparent post-2021 decline in the primary analysis reflects terminology-driven undercapture – post-2010 studies increasingly using “axial spondyloarthritis” in place of “ankylosing spondylitis” – together with citation and indexing lag; even within the expanded search, however, annual output declined modestly after 2021, consistent with a genuine plateau in the most recent years rather than a continued rise (Fig. 8).

**Figure 8.**
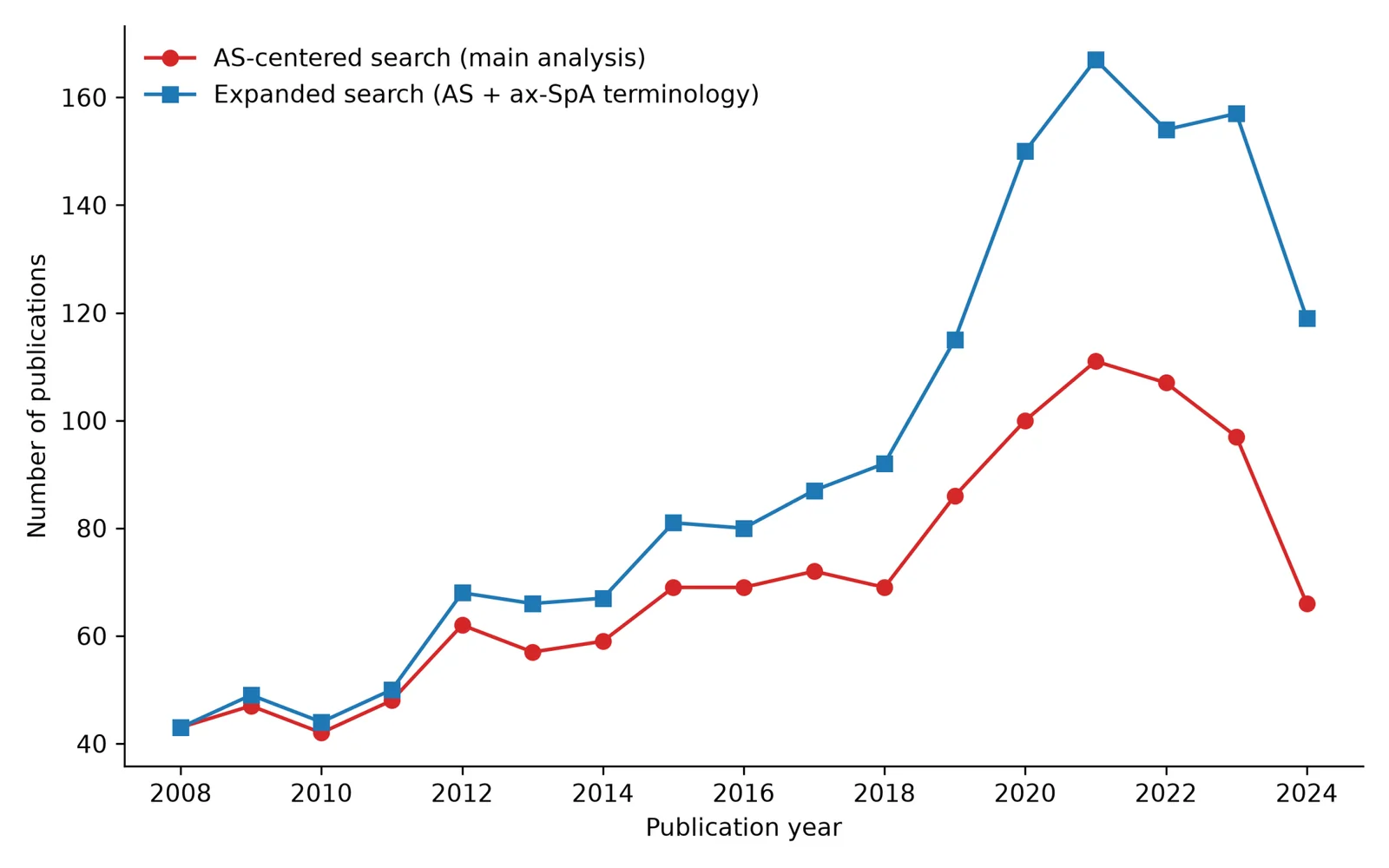
Sensitivity analysis: annual publication counts under the AS-only search versus the expanded search including axial spondyloarthritis terminology (2008–2024). AS = ankylosing spondylitis, ax-SpA = axial spondyloarthritis.

## 4. Discussion

### 4.1. Overview of the development in the diagnosis of ankylosing spondylitis

This study employed bibliometric methods to analyze articles related to AS diagnosis published since 2008, encompassing metrics such as publication volume, countries/regions of origin, authors and their affiliated institutions, journals, cited references, and keywords.

Over the past 17 years, 1205 AS diagnosis-related articles have been published. Based on annual publication trends, AS diagnostic research fell broadly into 2 phases: a growth period (2008–2021) and a transitional or reorientation period (2022–2024). The apparent decline in recent years is a recognized artifact of bibliometric review, reflecting citation lag, indexing delay, and COVID-era publication distortions,^[14]^ compounded by the incomplete 2024 data year, rather than evidence of intellectual stagnation. The top 3 journals contributing to this field were *Annals of the Rheumatic Diseases*, *Clinical Rheumatology*, and *Journal of Rheumatology. Annals of the Rheumatic Diseases*, a leading rheumatology journal, has published key guidelines from organizations such as ASAS and the European League Against Rheumatism. In 2024, *Clinical Rheumatology* and *Journal of Rheumatology* had impact factors of 2.9 and 3.6, respectively, aligning closely with the average annual citation rates for AS-related studies and underscoring their role as foundational journals in AS diagnosis research.

The advancement of diagnostic research is closely tied to a nation’s economic strength, research infrastructure, and sufficient patient populations. Publications from the top 10 most productive countries accounted for 70.62% of the total output, with 5 countries in Europe, 3 in Asia, and 2 in North America. Notably, 2 Asian countries – China and Turkey (both developing nations) – were ranked among these leaders. For instance, the prevalence of AS in China was 0.29% from 2005 to 2019,^[15]^ higher than the 0.11% reported in the Middle East during the same period but lower than the rates in the USA and Europe.^[16,17]^ This suggests that significant attention has been paid to AS diagnosis in China and Turkey. However, both countries lag behind North American and European nations in citation counts and international collaboration, in which the USA holds a dominant position. This reflects an imbalance between the quantity and quality of research output, highlighting the need for enhanced global cooperation and improved scientific capabilities. The most prolific author in AS diagnostic research is Joachim Sieper from Charité – Universitätsmedizin Berlin, a key contributor to ASAS clinical guidelines. High-productivity authors and institutions align with Germany’s central role in this field. Collaboration networks, however, also reveal the rising influence of Chinese researchers. For example, China Medical University and Liu Chong from The First Affiliated Hospital of Guangxi Medical University occupy central positions in these networks, underscoring China’s active engagement in international collaborations to advance AS diagnosis research.

The dual-map overlay of journals and the Three-Field Plot, based on keyword-author-keyword analysis, reveals the intellectual foundations of research in the AS-related field. Advances in understanding the pathophysiology of diseases rely on progress in foundational disciplines, whose outcomes gradually translate into clinical applications; AS is no exception. In the dual-map overlay, journals related to molecular, biological, and genetic research provide the basis for AS diagnosis, while journals focusing on sports rehabilitation, clinical nursing, and pharmaceuticals are also cited by AS diagnostic studies. From another perspective, high-impact scholars cite references encompassing disease-specific research (e.g., AS, ax-SpA, RA, and psoriatic arthritis) as well as broader themes, such as societal classification criteria, prevalence, and epidemiology.

### 4.2. Research hotspots in the diagnosis of ankylosing spondylitis

A review of the top 11 highly cited references revealed that most focused on imaging techniques for AS diagnosis. Imaging positivity remains critical for distinguishing AS within the spondyloarthritis (SpA) spectrum and differentiating it from other orthopedic conditions. Although X-rays have historically been used, recent studies advocate replacing conventional radiography with MRI to assess structural changes in the sacroiliac joints.^[18]^ MRI has emerged as a focal area in AS diagnostic research. In 2009, the ASAS/Outcome Measures in Rheumatology (OMERACT) MRI working group identified bone marrow edema or osteitis as essential for diagnosing active sacroiliitis.^[19]^ By 2010, the ASAS/OMERACT MRI study group highlighted anterior/posterior spondylitis and fat deposition at vertebral edges as key MRI markers for SpA.^[20]^ Subsequent studies, such as those by Komsalov et al, demonstrated the predictive value of MRI in early ax-SpA diagnosis, particularly through erosive changes in the sacroiliac joints in patients with preexisting bone marrow edema.^[21]^ Han et al further utilized MRI to detect hip inflammation in AS patients, noting that MRI-detected hip lesions often precede clinical symptoms.^[22]^ However, MRI alone cannot definitively diagnose AS. For instance, bone marrow edema may occur in healthy individuals, postpartum women, or patients with pelvic trauma.^[23]^ Integrating clinical features (e.g., HLA-B27 positivity and chronic back pain) with MRI findings improves diagnostic accuracy.^[24]^ A cohort study emphasized that repeated MRI scans in HLA-B27-positive males with chronic low back pain enhanced the reliability of ax-SpA diagnosis.^[25]^

From the timeline view of citation clusters, we observed the emergence of MR as a novel epidemiological method post-2021, which reemerged in subsequent thematic shifts. MR leverages genome-wide association studies (GWASs) to infer causal relationships between exposures and diseases using single nucleotide polymorphisms as instrumental variables. Recent MR studies have identified multiple risk factors for AS in European populations. For example, while gut microbiota, such as Bacteroides, Streptococcus, Proteobacteria, and Lachnospiraceae, showed no causal link to AS,^[25,26]^ higher abundances of Lactobacillaceae, Rikenellaceae, and Howardella were associated with reduced AS risk, whereas Actinobacteria and Ruminococcaceae NK4A214 correlated with increased risk.^[27]^ Additional gut microbiota-AS relationships have been proposed but are not elaborated here.

MR has also linked comorbidities, such as type 1 diabetes and acute iridocyclitis,^[28,29]^ to elevated AS risk. As GWAS data expand, MR is poised to uncover further AS risk factors, enhancing our understanding of its multifactorial etiology. It is important to clarify the interpretive level of these findings: MR primarily informs etiology and causality, not diagnosis. No current MR study provides a clinically actionable diagnostic criterion for AS; rather, MR findings serve as an etiological adjunct that may, over time, enable the discovery and prioritization of diagnostic biomarkers. Accordingly, we characterize MR as an emerging methodological trend in AS etiological research rather than as a diagnostic tool per se.

In the 2022 thematic keywords, biomarkers were highlighted, offering distinct advantages over clinical features and imaging evidence in differential diagnosis and early lesion detection. Preliminary studies have identified specific autoantibodies in AS. For instance, De Craemer et al demonstrated that anti-CD74 Immunoglobulin A antibodies distinguished AS from RA and fibromyalgia.^[30]^ Ziade et al further validated elevated anti-CD74 immunoglobulin A and IgG4 levels in patients with AS across Iraq and Lebanon.^[31]^ Feng et al discovered that serum anti-Kaiso autoantibodies differentiate non-radiographic ax-SpA (nr-axSpA) from AS, healthy controls, and RA, correlating positively with C-reactive protein levels and Bath Ankylosing Spondylitis Disease Activity Index scores, but inversely with disease duration.^[32]^ Ou et al developed a serum metabolite-based diagnostic model using metabolomics.^[33]^ AS comorbidities have spurred bioinformatics-driven investigations into shared pathogenic mechanisms. Examples include Prefoldin 5 and anti-Prefoldin 5 antibodies as biomarkers for AS-associated uveitis,^[34]^ Poly(A)-Specific Ribonuclease Subunit 3 (PAN3) as a genetic marker for AS with inflammatory bowel disease,^[35]^ and PAN3 as a shared biomarker for AS and ulcerative colitis.^[36]^ Given the unclear pathogenesis and pathological progression of AS, research on biomarkers holds significant potential for advancement. These biomarker findings, while promising, should be interpreted with caution. Most candidate biomarkers have not undergone external validation, show limited and variable sensitivity and specificity, and have not been standardized for clinical use; several derive from single cohorts or specific populations, raising concerns about reproducibility and population-specific bias. Few have been tested head-to-head against established diagnostic anchors such as MRI-detected sacroiliitis or HLA-B27 positivity. Until such validation is available, these markers should be regarded as research-stage leads rather than clinically deployable diagnostic tools.^[37]^

### 4.3. Transitional challenges and evolving terminology in the diagnosis of ankylosing spondylitis

From 2022 to 2024, the growth of AS diagnosis research appeared to plateau, a pattern we interpret as a transitional rather than stagnant phase. The cumulative annual publication curve across countries shows a leveling-off in most major contributing nations after 2021 (China being a partial exception), consistent with citation lag, indexing delay, COVID-era publication distortions, and the incomplete 2024 data year rather than a genuine loss of research momentum.

This may be linked to the conceptual interchange between AS and radiographic axial spondyloarthritis (r-axSpA). In 2023, the ASAS explored the interchangeability of diagnosing AS using the New York criteria and r-axSpA under the ASAS criteria, proposing to replace “AS” with “r-axSpA.”^[38]^ The coexistence of these 2 terms has created challenges in diagnostic classification. Even though the ASAS advocated for this conceptual shift, widespread acceptance of the new terminology among researchers requires time. The decline in publications on AS diagnosis may be related to this updated terminology.

It is also noteworthy that AS involves highly complex pathophysiological mechanisms. Current evidence supports both auto-inflammatory pathways, such as the roles of gut microbiota and joint trauma in AS pathogenesis, and autoimmune mechanisms, including dysregulated autoantibodies and T cell subsets.^[39]^ Additionally, AS exhibits substantial unexplained genetic heterogeneity, with significant ethnic disparities that cannot be overlooked. All current MR studies on AS are derived from European GWAS, leaving uncertain the applicability of these findings to Asian and other populations. Furthermore, the clinical utility of bioinformatic analyses based on public databases remains questionable in practical diagnostic and therapeutic settings.

Finally, significant gaps remain in AS diagnostic research. Burst detection of keywords identified “children” as an emerging focus post-2021. Among SpA subtypes, juvenile spondyloarthritis (JSpA) may progress to AS, particularly in cases with enthesitis-related pathological manifestations. Researchers have applied imaging techniques to evaluate JSpA, demonstrating MRI’s superiority over X-ray.^[40]^ Some studies have also investigated MRI threshold values for JSpA diagnosis,^[41]^ though these imaging findings have yet to establish a clinical consensus, warranting further investigation. Notably, psoriatic arthritis patients exhibit axial inflammatory manifestations that require differentiation from AS patients with secondary psoriasis. German researchers have identified distinct patterns of joint inflammation between these groups^[42]^; however, detailed clinical characterization remains incomplete. Emerging studies have explored psychiatric dimensions of AS. Two prospective cohort studies consistently demonstrated that intense emotional events transiently increase disease activity in patients with AS,^[43,44]^ while certain mental disorders, such as schizophrenia, have been identified as risk factors for AS development. However, psychiatric manifestations lack specificity for clinical identification of AS patients, limiting their diagnostic utility. Taken together, the bibliometric patterns described here – the shift toward MRI-based assessment, the search for serological and metabolic biomarkers, and the ongoing redefinition of the diagnostic construct itself – reflect, and in turn help explain, the persistent clinical diagnostic uncertainty and delay that continue to burden patients with ax-SpA. A bibliometric perspective thus offers not only a map of the field but also a window onto the clinical challenges that motivate it.

## 5. Conclusion

Current AS diagnostic research has transitioned from a growth phase (2008–2021) to a transitional or reorientation period (2022–2024). While Germany remains influential, China shows growing potential. Key focuses include MRI analysis, MR, and biomarkers. Current challenges involve conceptual shifts in AS/r-axSpA terminology, narrow research perspectives, and insufficient diagnostic-specific clinical indicators.

## 6. Limitations

Several limitations should be acknowledged. First, the ongoing conceptual transition between AS and SpA terminology hampers the comprehensive inclusion of all relevant diagnostic studies: many post-2010 studies deliberately avoid the term “ankylosing spondylitis” in favor of “radiographic axial spondyloarthritis,” although some publications explicitly reference both concepts.^[45]^ Even so, our AS-centered search may systematically undercapture recent r-axSpA diagnostic research. Our sensitivity analysis comparing trends with and without explicit AS terminology confirms this undercapture (384 additional publications) but also shows that the main conclusions are robust to the terminology choice. Second, our interpretation of the post-2021 period as a transitional or reorientation phase is subject to bibliometric time-lag bias because recently published articles have had less time to accumulate citations and be indexed, and the 2024 data year is incomplete; the trend, therefore, primarily reflects publication counts rather than intellectual stagnation. Finally, our exclusive focus on English-language publications, while standard for bibliometric studies, may omit contributions published in other languages, including German-language research, given Germany’s central role in this field.

## Acknowledgments

We would like to express our sincere gratitude to the lecturer of the Bibliometrics course and the Dingxiangyuan Network Platform.

## Author contributions

**Data curation:** Zhihao Qiu, Keming Chen, Zijie Chen.

**Formal analysis:** Zhihao Qiu, Zijie Chen.

**Investigation:** Zhihao Qiu, Ruibo Xia, Zhe Yang, Zijie Chen.

**Project administration:** Kepeng Yang.

**Supervision:** Kepeng Yang.

**Validation:** Ruibo Xia, Zhe Yang.

**Writing – original draft:** Zhihao Qiu.

**Writing – review & editing:** Kepeng Yang.
